# Coordination of Fictive Motor Activity in the Larval Zebrafish Is Generated by Non-Segmental Mechanisms

**DOI:** 10.1371/journal.pone.0109117

**Published:** 2014-10-02

**Authors:** Timothy D. Wiggin, Jack H. Peck, Mark A. Masino

**Affiliations:** 1 Graduate Program in Neuroscience, University of Minnesota, Minneapolis, Minnesota, United States of America; 2 Department of Neuroscience, University of Minnesota, Minneapolis, Minnesota, United States of America; Institut Curie, France

## Abstract

The cellular and network basis for most vertebrate locomotor central pattern generators (CPGs) is incompletely characterized, but organizational models based on known CPG architectures have been proposed. Segmental models propose that each spinal segment contains a circuit that controls local coordination and sends longer projections to coordinate activity between segments. Unsegmented/continuous models propose that patterned motor output is driven by gradients of neurons and synapses that do not have segmental boundaries. We tested these ideas in the larval zebrafish, an animal that swims in discrete episodes, each of which is composed of coordinated motor bursts that progress rostrocaudally and alternate from side to side. We perturbed the spinal cord using spinal transections or strychnine application and measured the effect on fictive motor output. Spinal transections eliminated episode structure, and reduced both rostrocaudal and side-to-side coordination. Preparations with fewer intact segments were more severely affected, and preparations consisting of midbody and caudal segments were more severely affected than those consisting of rostral segments. In reduced preparations with the same number of intact spinal segments, side-to-side coordination was more severely disrupted than rostrocaudal coordination. Reducing glycine receptor signaling with strychnine reversibly disrupted both rostrocaudal and side-to-side coordination in spinalized larvae without disrupting episodic structure. Both spinal transection and strychnine decreased the stability of the motor rhythm, but this effect was not causal in reducing coordination. These results are inconsistent with a segmented model of the spinal cord and are better explained by a continuous model in which motor neuron coordination is controlled by segment-spanning microcircuits.

## Introduction

Locomotion in vertebrates is organized by spinal neural circuits called central pattern generators (CPGs) that are capable of driving patterned motor neuron output even in the absence of patterned synaptic input [1]. Despite their importance, the cellular basis for most vertebrate CPGs is still incompletely characterized [2], [3]. Because there is evidence for evolutionary conservation of CPG elements such as cell types [4], two vertebrate species with characterized locomotor CPGs, lamprey [5] and tadpole [6], are frequently used as the basis of models of locomotor CPGs in other animals [7] (but see also [8]).

One model of the vertebrate spinal locomotor CPG proposes that it is composed of a series of reiterated circuits with connecting projections [7], [9]. This segmental CPG model is comparable to the known organization of several invertebrate locomotor CPGs. For example, in crayfish and leech the locomotor CPGs are composed of segmentally reiterated groups of neurons with local connections that coordinate alternation in antagonist motor neurons and with longer range projections that drive rostral to caudal propagation [10]–[12]. The segmental CPG model is supported also supported by experimental evidence in vertebrates. Lesion studies have demonstrated that rhythmic, coordinated motor output can be evoked using tonic excitatory drive from as few as 2 spinal intact segments in chicks [13], rats [14], lamprey [15], and salamanders [16]. Complementing this experimental evidence, computational models of the lamprey locomotor CPG that use spinal segments as the units of circuit reiteration accurately reproduce swimming output [5], [17].

One deficiency in the segmental CPG model is that there is little anatomical evidence for segmental distribution of interneurons that would make up the segmental CPGs [18]. An alternative is a continuous model of the vertebrate spinal locomotor CPG, which is proposed as an unsegmented, continuous collection of neurons with gradients of soma and synaptic density that drive appropriately timed motor output. Based on anatomical distributions of neurons, quantitative continuous models of the lamprey [19] and tadpole [20], [21] locomotor systems have been developed that produce swimming-like motor output. Continuous CPG models of rhythm generation have also been proposed for the mammalian locomotor circuit based on the properties of motor deletions in fictive locomotion [22].

The larval zebrafish is a useful model of locomotion because it has well developed genetic tools and shares many genetic markers of cell type with mammals, but has a simpler motor output than quadruped locomotion [23]. Larval zebrafish swim in a “beat-and-glide” pattern composed of brief episodes of active swimming separated by periods of inactivity [24]. During each episode, the larvae undulate using side-to-side lateral alternation and rostrocaudal progression of the body wave [25], [26]. Fictive swimming in both intact and spinalized larval zebrafish retains the episodic nature of free-swimming larvae [27], [28]. Within each episode, bursts are produced along the body with a rostrocaudal delay and bursts alternate on each side of the body [27]. These burst-timing relationships drive the undulatory movement of the free-swimming larvae, and throughout this paper we refer to them collectively as “coordination.” In a previous study we demonstrated that the production of episodes of activity in larval zebrafish depends upon a distributed spinal circuit, and that rostrocaudal delay and side-to-side alternation are independent of episode production [29]. In this study, we tested the hypothesis that larval zebrafish have segmentally reiterated locomotor circuits for production of rostrocaudal delay and side-to-side alternation. We found that the motor output of the spinal cord following transection or reduction of inhibitory synaptic strength is not consistent with a segmental CPG model, and is more consistent with a continuous CPG model.

## Methods

### Ethics Statement

All procedures were approved by the Animal Care and Use Committee of the University of Minnesota Twin Cities, Approval #1305-30622A.

### Animals and solutions

Wild type adult zebrafish (*Danio rerio*, Segrest Farms, Gibsonton, FL) were maintained in the University of Minnesota Zebrafish Core Facility. Group breeding tanks of adult zebrafish were set up daily to produce clutches of embryos with timed fertilization between 8:45 and 9:00am. Embryos and larval zebrafish were maintained in 100 mm petri dishes filled with embryo water (60 µg/ml Instant Ocean salt mix, Cincinnati, OH) and 0.0002% methylene blue in a 28.5°C incubator with a 14:10 light:dark cycle. All experiments were carried out using larval zebrafish 4 to 6 days post fertilization (dpf). At this age, the sex of the larvae is not determined [30]. Chemicals and drugs were obtained from Sigma-Aldrich Chemical (St. Louis, MO), unless otherwise noted. Zebrafish extracellular saline was composed of (in mM): 134 NaCl, 2.9 KCl, 1.2 MgCl_2_, 2.1 CaCl_2_, 10 HEPES buffer, 10 glucose, adjusted to pH 7.8 with NaOH and 300 mOsm with sucrose [31], [32].

### Peripheral Nerve Recordings

Larval zebrafish were prepared for peripheral nerve (PN) recordings as previously described [27]. Briefly, larval zebrafish were anesthetized with 0.02% Tricaine-S (Western Chemical, Ferndale, WA) in extracellular saline, pinned in a Sylgard-lined dissecting dish, and skin was removed from the regions of the body to be recorded. Larvae were paralyzed using 5 µl of 0.1 mM α-bungarotoxin (Tocris, Ellisville, MO) added to the small volume (∼15 µL) of extracellular saline in the dissection dish. Paralyzed larvae were transected while bathed in extracellular saline using a razor blade shard to completely sever the spinal cord and overlying muscle (razor blade: FA-10 Feather S, Ted Pella, Redding, CA). Transections nicked, and occasionally severed, the notochord and completely separated the musculature, including the dorsal muscle. Larvae were allowed to recover for 20–30 minutes following transection and prior to peripheral nerve recordings. Larvae used for unilateral PN recordings (to measure rostrocaudal delay) were pinned with one side of the larva facing up. Larvae used for bilateral PN recordings (to measure side-to-side alternation) were rotated into a dorsal-up position so that both sides of the larva were accessible. Larvae were continuously superfused with extracellular saline during all recordings.

### Experimental Groups

In this study we used a range of reduced spinal cord preparations of larval zebrafish. Spinalized larvae and reduced preparations only produced fictive motor output when it was evoked by NMDA. NMDA was superfused for approximately 20 minutes prior to the beginning of PN recordings, and continued throughout the recording. The NMDA concentration used was 100 µM unless otherwise noted.

The experimental conditions were as follows: 1) Spinalized preparations were transected at body segment 3 (S3) to separate the spinal cord from the hindbrain. 2) Rostral-10 preparations were transected at S3 and S14, leaving 10 intact segments between the transection sites centered on S8. 3) Rostral-5 preparations were transected at S5 and S11, leaving 5 intact segments centered on S8. 4) Middle-10 preparations were transected at S6 and S17, leaving 10 intact segments centered on S11. 5) Middle-5 preparations were transected at S10 and S16, leaving 5 intact segments centered on S13. 6) Caudal-5 preparations were transected at S16 and S22, leaving 5 intact segments centered on S19.

Segments were counted using the anal pore as the marker for the ventral side of S15 and the first visible segment caudal to the head as S1. Because of ambiguity in determining the location of body segment landmarks between larvae, it is likely that the borders of these transected regions were offset rostrally or caudally by up to 1 body segment, but the number of body segments was consistent between dissections. All experimental groups contained larvae from at least 2 clutches.

### Electrophysiology

PN recordings were performed as previously described [27]. Briefly, larvae were placed on the stage of an upright microscope (Olympus BX51 WI, Center Valley, PA), and continuously superfused with extracellular saline at room temperature (20–22°C). PN recordings were obtained using glass suction electrodes with tip sizes ranging from 9 to 15 µm. Recordings of laterally mounted larvae were obtained from the intermyotomal cleft adjacent the horizontal septum; paired recordings of dorsoventrally mounted larvae were obtained from the intermyotomal clefts on opposite sides of the larvae. Signals were obtained using an Axon Instruments Multiclamp 700B amplifier and acquired with an Axon Instruments Digidata 1440A controlled by pClamp 10 software (Molecular Devices, Union City, CA).

### Analysis of Peripheral Nerve Recordings

We used a custom Matlab (Mathworks, Natick, MA) program developed in our laboratory to detect fictive swimming in PN recordings automatically, as previously described [29]. Briefly, for each voltage sample (*v(n)*), the voltage autocorrelation (*c_n_(k)*) was computed over a small window (3 ms) centered at *v(n)*. A subset of the autocorrelation values were used to compute a test-statistic (*c_n_*) for each *v(n)*, where *c_n_* is the sum of the *c_n_(k)* in the range *k* = [1], [2]. This range of *k* was chosen empirically to optimize burst detection and noise rejection in low amplitude recordings. Activity was considered present at *v(n)* when the test statistic was greater than a detection threshold *T*. *T* was set for each recording as the maximum value of the test statistic in a region of the recording that was visually inspected and confirmed to not contain any bursting activity (typically the first second of the recording). If the test statistic remained above threshold for at least 3ms, the supra-threshold voltage samples were identified as a burst.

We defined the properties of fictive motor output as follows: Burst Duration was the time between the test statistic rising above threshold and falling below threshold. Burst Frequency was the inverse of the mean inter-burst period (IBP), which was defined for each pair of bursts as the time from the midpoint of the first burst to the midpoint of the second burst. IBPs longer than 200ms were excluded from quantification because, in unperturbed swimming, these IBPs are times between episodes rather than part of the intra-episode locomotor rhythm. This IBP threshold is longer than would be strictly necessary based on typical zebrafish behavior in order to accommodate the phenomenon of “missed” bursts. A “missed” burst is a time when a burst is expected based on the locomotor rhythm, but not detected by the PN recording. We do not have evidence that missed bursts reflect changes in the underlying behavior, instead we believe that they are due to under-sampling the motor pool. In order to avoid inappropriately partitioning activity into separate episodes because of missed bursts, we use an IBP threshold twice as long as would be sufficient if we assumed perfect burst detection.

### Phase Vector Sum Analysis

**Figure 1:**
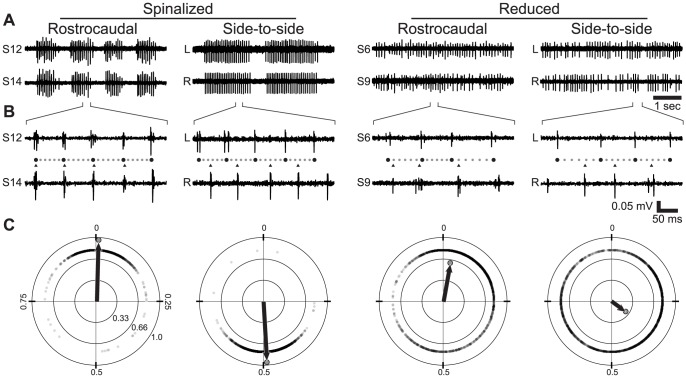
Phase Vector Sum Analysis of Fictive Motor Output. (A) PN recordings of fictive motor output produced by representative spinalized larvae (left traces) and reduced spinal cord preparations (right traces). Left (L) and right (R) sides of the body are indicated in traces showing side-to-side alternation. The rostrocaudal location of each recording is indicated by segment number (e.g. S12) in traces showing rostrocaudal progression. (B) The indicated regions of the top traces at a finer time scale. The relative phases of the bursts in the paired recordings are illustrated using circles (phase markers) and triangles (follower burst times). Black circles indicate burst times, smaller gray circles divide each burst period into 6 equally long intervals. (C) Polar plots showing the phase of each burst (small gray circles) from the follower recording site relative to bursts from the leader recording site, for each group indicated above the plot. Individual burst phases are plotted at an arbitrary radius for illustrative purposes, they are treated as unit vectors when calculating vector sums. Concentric circles are plotted at distances of 0.33, 0.66 and 1.0 from the fixed point; cross-hairs separate the quadrants. The normalized vector sums of the bursts from each representative preparation are illustrated by an arrow and a large gray circle at the terminal point.

We used a phase-based analysis to quantify the changes in rostrocaudal delay and side-to-side alternation (Fig. 1). One PN recording was *a priori* designated as the phase leader and the other as the phase follower. For bilateral recordings, the left side of the animal was the leader, and in unilateral recordings of rostrocaudal delay, the more rostral recording site was the leader. In the phase leader recording, the IBP of each pair of chronologically adjacent bursts was calculated. Burst pairs with an IBP greater than 2× the mean of that record were excluded from further analysis because of the possibility of missed bursts distorting the phase calculation. The time period between each pair of bursts in the leader recording was checked for the presence of a burst in the phase follower recording (Fig. 1A,B). If one or more follower bursts were present, the phases (in radians) were calculated as 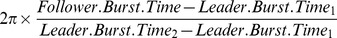, where subscripts 1 and 2 indicate the initial and final bursts of the pair of bursts in the leader recording, respectively. After the follower phases were calculated, they were converted into unit vectors in a polar plane (Fig. 1C). Standard vector addition was used to calculate the vector sum of all burst phase vectors, and the magnitude of the resulting vector sum was divided by the total number of phase vectors, normalizing it to the range 0–1. Finally, the phase of the vector sum was divided by 2π, converting from radians into the range 0–1. This process produced a single mean phase vector for each preparation that has two parameters: 1) *θ*, the phase angle of the vector, a measure of mean phase offset between the leader and follower. 2) *r*, the vector magnitude, a quantification of the degree of consistency of the phases of each follower burst relative to the leader (Fig. 1C). For rostrocaudal recordings, the phase was divided by the number of segments separating the recording sites, yielding phase lag per body segment.

### Statistical Analysis

Measurement of the variability of burst period and rostrocaudal delay is necessary to quantify the stability of the motor rhythm and the reliability of rostrocaudal delay, respectively. Variance and standard deviation are both sensitive to the effects of outliers, so we chose a more robust statistic for comparing the variability of groups to one another: the Median Absolute Deviation (MAD) [33]. Tests for significant differences in episode and burst properties, the magnitude of the phase vector and MAD values were carried out using one-, two- and three-way ANOVAs and subsequent protected *t*-tests. The value and deviation of the phase angle for each group was calculated using circular statistics, and the Watson-Williams test was used to test for significant differences between groups. Statistical tests were carried out using SigmaPlot 12 software (SyStat Software, San Jose, CA), Microsoft Excel (Microsoft, Seattle, WA), or the Matlab CircStat toolbox [34]. An α level of 0.05 was used to determine statistical significance. Linear data are expressed as the mean and standard deviation, phase data are expressed as the circular mean with angular variation.

## Results

### NMDA Induces Non-Episodically Organized Fictive Motor Activity in the Reduced Larval Zebrafish Spinal Cord

**Figure 2:**
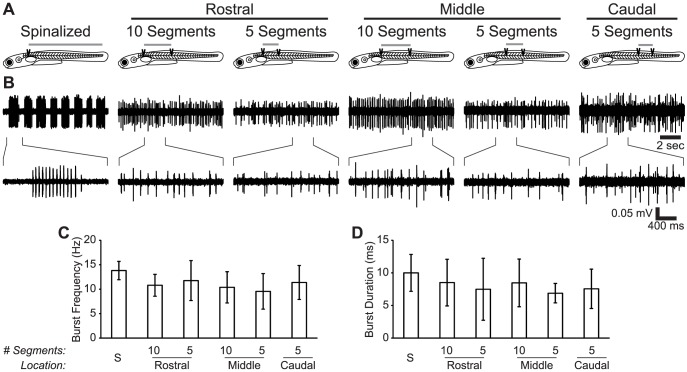
NMDA Induces Fictive Motor Activity in Spinalized and Reduced Larval Zebrafish. (A) Schematic diagrams of Spinalized, Rostral-10, Rostral-5, Middle-10, Middle-5, and Caudal-5 preparations. Dark wedges indicate the sites of spinal transections, and gray bars above each larva indicate the spared spinal cord region used for recordings subsequent to transection. (B) Representative traces showing fictive motor activity in each of the experimental conditions below its respective schematic diagram. Bottom traces show the indicated region at a finer time scale. (C–D) Plots of burst frequency (C) and burst duration (D) in each experimental group. The bar labeled “S” is the spinalized group.

The only published method for inducing fictive locomotion in spinalized zebrafish larvae and reduced larval zebrafish spinal cords is bath application of NMDA [28], [29], [35]. The episodic character of intact and spinalized fictive locomotion is disrupted by spinal transections in a graded fashion depending upon the quantity and region of spared spinal cord [29]. In this study, we used small isolated regions of the spinal cord (5 or 10 segments, approximately 15% or 30%, respectively, of the full cord; Fig. 2A). NMDA (100 µM) induced fictive locomotion in these reduced preparations was qualitatively different from spinalized swimming because it lacked episodic structure (Fig. 2B). We quantified the parameters of fictive motor output from the following experimental groups (described in *Methods*): Spinalized (*n* = 11) Rostral-10 (*n* = 9), Middle-10 (*n* = 8), Rostral-5 (*n* = 6), Middle-5 (*n* = 6), Caudal-5 (*n* = 6). Burst frequency and burst duration did not vary between the groups (One-way ANOVAs; all *F*_(5,40)_<2.17; all *p*>0.07; Fig. 2C,D).

### Rostrocaudal Phase Consistency and Motor Rhythm Stability are Impaired in Reduced Spinal Cord Preparations

**Figure 3:**
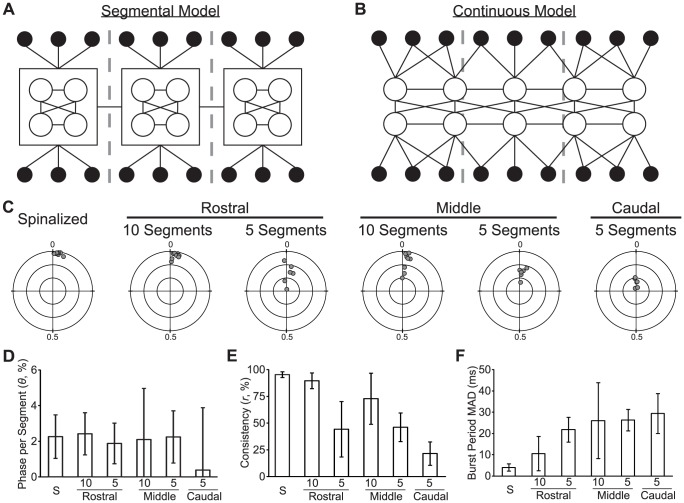
Rostrocaudal Phase Consistency and Motor Rhythm Stability are Decreased by Spinal Transection. (A–B) Diagrams of segmental (A) and continuous (B) models of the spinal locomotor CPG. The models show the spinal circuit with the rostrocaudal axis horizontal and the mediolateral axis vertical. Body segment boundaries are shown with gray dashed lines. In both models, each segment contains two groups of motor neurons (black-filled circles), each group innervating one lateral hemi-segment. In both models, interneurons (open circles) form synaptic connections (black lines) with each other and with motor neurons. In the segmental model (A), each segment contains a reiterated interneuron circuit that controls local motor neurons and communicates with other segmental circuits. In the continuous model (B), interneurons are distributed and form synaptic connections based on inter-somatic distance and are independent of segmental boundaries. In both models, patterns of interneuron connectivity are strictly illustrative and should not be interpreted as definite synaptic connections between defined interneurons. (C) Polar plots showing the normalized rostrocaudal phase vector sum (gray circle) of each preparation in the experimental group indicated above the plot. Concentric circles are plotted at distances of 0.33, 0.66 and 1.0 from the fixed point; cross-hairs separate the quadrants. (D–F) Plots of mean phase delay per segment (D), mean phase consistency (E), and mean burst period MAD (F) of each experimental group. The bar labeled “S” is the spinalized group. Significant differences are not indicated due to the number of pair-wise comparisons (see text in *Results*).

A segmental model of the spinal locomotor network (Fig. 3A) would predict that the phase relationship between the fictive motor outputs of two segmental CPGs should depend on the connections (direct or indirect) between the segments and the state of each segmental CPG. In contrast, a non-segmental model of the locomotor network (Fig. 3B) would predict that the coordination of fictive motor output at two points along the rostrocaudal axis of the larva would depend upon the integrity of the entire circuit. To determine if rostrocaudal delay is affected by reducing the number of contiguous spinal segments surrounding the PN recordings, we performed two-point unilateral PN recordings of fictive motor output produced by isolated regions of the larval zebrafish spinal cord (Spinalized, *n* = 9; Rostral-10, *n* = 9; Middle-10, *n* = 8; Rostral-5, *n* = 6; Middle-5, *n* = 6; Caudal-5, *n* = 6; Fig. 3C). We measured coordination of the motor output using phase vector sum analysis, and we measured the stability of the motor rhythm using the median absolute deviation (MAD) of the burst period (see *Methods*). PN recordings were performed 1 to 4 segments apart (mean: 2.16(SD 0.75)); phase delay was normalized to phase per segment. Mean rostrocaudal phase delay per segment did not differ among the experimental groups (Watson-Williams test; *F*_(5,37)_ = 0.77; *p* = 0.58; Fig 3D). However, there was a significant effect of experimental group on phase consistency (One-way ANOVA; *F*_(5,37)_ = 23.2; *p*<0.001; Fig 3E). Post-hoc tests revealed that spinalized larvae had higher phase consistency than all groups but Rostral-10 (Corrected *t*-tests; all *t*>2.8; all *p*<0.04), that 10 segment experimental groups had higher phase consistency than 5 segment experimental groups (Corrected *t*-tests; all *t*>3.1; all *p*<0.025), and that there were no significant differences among the 5 segment experimental groups. The trends revealed by the post-hoc tests were confirmed by a two-way ANOVA of only the Rostral-10, Rostral-5, Middle-10, and Middle-5 experimental groups that showed a significant main effect of number of segments (*F*_(1,25)_ = 26.2; p<0.001), but no main effect of location of segments or interaction of number of segments and location (all *F*_(1,25)_<1.8; all *p*>0.19). There was also a significant effect of experimental condition on the burst period MAD (One-way ANOVA; *F*_(5,37)_ = 8.1; *p*<0.001; Fig. 3F). Post-hoc tests revealed that spinalized larvae had lower burst period variability than all other groups but Rostral-10 (Corrected *t-*tests; all *t*>3.4; all *p*<0.018). Rostrocaudal coordination of fictive motor output is impaired in reduced larval zebrafish spinal cord. The number of spared segments, but not the rostrocaudal location of the segments, determines the degree of impairment. This effect may be either due to injuring a distributed coordination circuit or due to unstable oscillation of segmental CPGs.

### Side-to-Side Phase Consistency and Motor Rhythm Stability are Impaired in Reduced Spinal Cord Preparations

**Figure 4:**
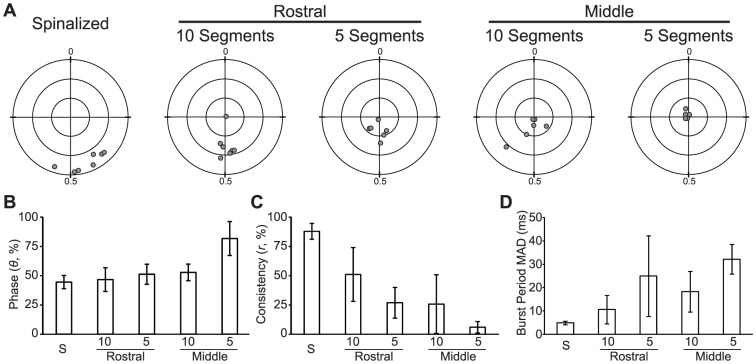
Side-to-Side Phase Consistency and Motor Rhythm Stability are Decreased by Spinal Transection. (A) Polar plots showing the normalized contralateral phase vector sum (gray circle) of each preparation in the experimental group indicated above the plot. Concentric circles are plotted at distances of 0.33, 0.66 and 1.0 from the fixed point; cross-hairs separate the quadrants. (B-D) Plots of mean phase offset (B), mean phase consistency (C), and mean burst period MAD (D) of each experimental group. The bar labeled “S” is the spinalized group. Significant differences are not indicated due to the number of pair-wise comparisons (see text in *Results*).

A segmental CPG model of the spinal locomotor network (Fig. 3A) would predict that phase relationships between the contralateral sides of the same segment should depend only on the operation of each segmental circuit and not on inter-segmental connections. Alternatively, a continuous CPG model of the spinal locomotor network (Fig. 3B) would predict that the coordination between contralateral sides of a single segment of the larva would depend upon the integrity of the entire circuit. To determine if side-to-side alternation is affected by reducing the number of contiguous spinal segments, we performed two-point bilateral PN recordings of fictive motor output produced by isolated regions of the larval zebrafish spinal cord (Spinalized, *n* = 7; Rostral-10, *n* = 7; Middle-10, *n* = 6; Rostral-5, *n* = 6; Middle-5, *n* = 6; Fig. 4A). We measured coordination of the motor output using the phase vector sum analysis, and we measured the stability of the motor rhythm using the MAD of the burst period. There was a significant effect of experimental group on side-to-side phase (Watson-Williams test; *F*_(4,27)_ = 7.1; *p*<0.001; Fig 4B). This difference was driven by the Middle-5 group, which differed significantly from the spinalized and Rostral-10 larvae (Corrected Watson-Williams test; all *p*<0.025). The Middle-5 larvae had extremely low phase consistency (Fig. 4C), and the mean phase of this group is likely not functionally meaningful. There was also a significant effect of experimental group on phase consistency (One-way ANOVA; *F*_(4,27)_ = 23.2; *p*<0.001; Fig 4C). Post-hoc tests revealed that spinalized larvae were more consistent than all of the experimental groups of reduced spinal cord preparations (Corrected *t*-tests; all *t*>4.1; all *p*<0.002). A two-way ANOVA of Rostral-10, Rostral-5, Middle-10, and Middle-5 groups showed significant main effects of both the position of segments (*F*_(1,21)_ = 9.5; p = 0.006), and the number of segments (*F*_(1,21)_ = 8.7; p = 0.008) with no significant interaction. There was also a significant effect of experimental condition on the burst period MAD (One-way ANOVA; *F*_(4,27)_ = 9.0; *p*<0.001; Fig. 4D). Post-hoc tests revealed that spinalized larvae had lower burst period variability than 5 segment transected preparations (Corrected *t*-tests; all *t*>3.9; all *p*<0.004). Side-to-side alternation of fictive motor output is impaired in reduced zebrafish spinal cord and both the number of spared segments and the rostrocaudal location of the segments determines the degree of impairment. There is also a significant decrease in the stability of the motor rhythm in the reduced spinal cord conditions. The decrease in coordination may be due to either injuring a distributed coordination circuit or to unstable oscillation of hemisegmental CPGs.

### Side-to-Side Alternation is Impaired More than Rostrocaudal Delay in Reduced Spinal Cord Preparations

To compare the effect of spinal cord reduction on rostrocaudal and side-to-side coordination, we performed a three-way ANOVA (recording type X number of segments X rostrocaudal location) on the phase consistency of the reduced spinal cord experimental groups shared between the unilateral and bilateral PN recording experiments (Rostral-10, Rostral-5, Middle-10, and Middle-5; Figs. 3,4). Spinal transections impaired the phase consistency of side-to-side alternation significantly more than the rostrocaudal delay (*F*_(1,46)_ = 48.5; *p*<0.001). Consistent with the two-way ANOVA results, rostral segments produced more consistent phase delays than middle segments and 10 segment regions of contiguous spinal segments produced more consistent phase delays than 5 segment regions (all *F*_(1,46)_>8.8; all *p*<0.005), but there were no significant interactions. There were no significant differences between the burst period MAD recorded in unilateral and bilateral experiments (Two-way ANOVA; *F*_(1,59)_ = 0.03; *p* = 0.86).

### Both Intra-segmental and Inter-segmental Coordination are Disrupted by Spinal Transections

The preceding experiments were not sufficient to exclude either the segmental or continuous model of the spinal CPG. The graded disruption of coordination observed in progressively reduced larval zebrafish spinal cord would be predicted by a continuous CPG model, but could also be explained by the disruption of rhythm generation in putative segmental CPGs. However, the models make different predictions about the effect of transection on the fictive motor output of an individual segment. The segmental model (Fig. 3A) predicts that the coordination of output from a single motor pool would be unaffected by spinal transections or decreased motor rhythm stability. On the other hand, the distributed CPG model (Fig. 3B) does not require that motor neurons within a segment have any more shared drive or synchronous output than motor neurons in different segments.

**Figure 5:**
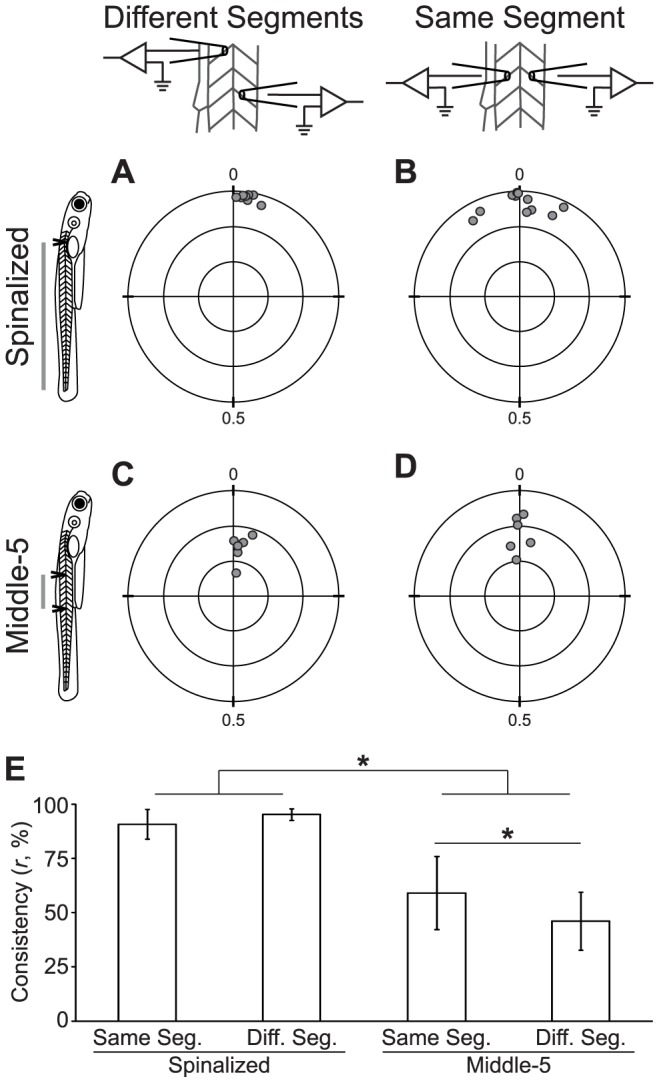
Both Intra-segmental and Inter-segmental Coordination are Disrupted by Spinal Transections. (A–D) Polar plots of the normalized phase vector sum (gray circles) of Spinalized (A,B) and Middle-5 (C,D) preparations recorded at two locations at a rostrocaudal offset (A,C) or on the same body segment (B,D). Concentric circles are plotted at distances of 0.33, 0.66 and 1.0 from the fixed point; cross-hairs separate the quadrants. (E) Plot of mean vector sum consistency (*r*) of the four experimental groups above. * Statistically significant difference.

To test the hypothesis that coordination within a segment is unaffected by spinal transection, we compared the timing of fictive motor output from two points on a single hemi-segment and from two ipsilateral hemi-segments in reduced spinal cord preparations (Fig. 5). We performed same segment ipsilateral PN recordings on spinalized (*n* = 11; Fig. 5B) and Middle-5 larvae (*n* = 6; Fig. 5D). Data from different-segment recordings of spinalized and Middle-5 larvae presented in Fig. 3 are reproduced here for purposes of comparison (Fig. 5A,C). There is a greater spread of mean phase in the spinalized same-segment recordings than in the spinalized different-segment recordings (Fig. 5A,B). The greater spread is due to the different-segment mean phase being divided by the number of segments between the recordings (see *Methods*), an operation that does not apply to same-segment recordings. There was a significant main effect of spinal cord reduction on phase consistency (Two-way ANOVA; *F*_(1, 27)_ = 113.9; *p*<0.001; Fig. 5E), consistent with our previous results (Fig. 3). There was no main effect of recording in the same-segment versus different segments on phase consistency (Two-way ANOVA; *F*_(1, 27)_ = 1.3; *p* = 0.27; Fig. 5E), but a post-hoc test did show that Middle-5 same segment recordings are significantly more coordinated than Middle-5 different segment recordings (Corrected *t*-test; *t* = 2.2; *p* = 0.04). These results indicate that motor neuron coordination within a body segment is significantly reduced by spinal transection, which is inconsistent with the segmental CPG model. The small difference in coordination between Middle-5 same segment and Middle-5 different segment recordings could be due to weak intra-segmental coupling of the motor neurons or a bias toward shared synaptic input within a segment [36].

### Reduced Synaptic Inhibition Reversibly Reduces Coordination and Burst Period Stability

**Figure 6:**
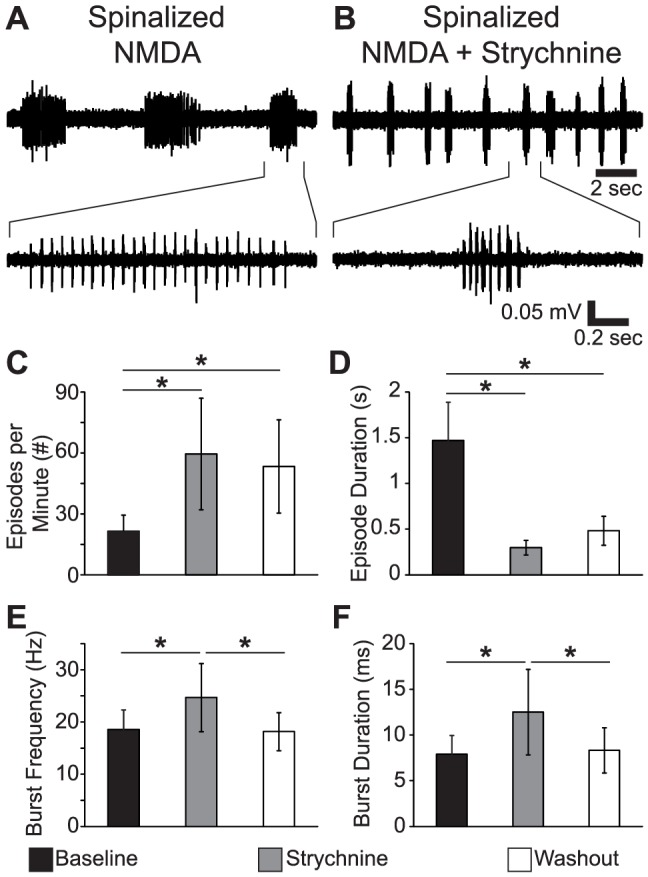
Reduced Synaptic Inhibition Perturbs Spinalized Fictive Motor Output Without Disrupting Episodic Structure. (A–B) Representative traces of the fictive motor output from a representative spinalized larva in 50 µM NMDA (A) and following application of 1 µM Strychnine (B). Top traces are episodes of fictive motor activity; bottom traces show bursts in the indicated regions at a finer time scale. (C–F) Plots of episode frequency (C), episode duration (D), burst frequency (E) and burst duration (F) in Baseline (50 µM NMDA), Strychnine (50 µM NMDA, 1 µM Strychnine), and Washout (50 µM NMDA) conditions. * Statistically significant difference.

Transection experiments allowed us to measure the effect of reducing ascending and descending synaptic input on the coordination of fictive motor output. These lesions disrupt episodic organization and are irreversible. Strychnine, a glycine receptor antagonist, has been demonstrated to reduce swimming speed without reducing tail beat frequency or disrupting episodic organization, presumably through weakening each cycle of swimming [37]. In order to determine if fictive motor coordination can be disrupted independently of disrupting episodic organization, we pharmacologically suppressed inhibitory neurotransmission with strychnine. We used a strychnine concentration (1 µM) that has been shown to significantly decrease glycinergic neurotransmission [38]. The combination of 100 µM NMDA and 1 µM strychnine evoked fictive motor output in which adjacent bursts fused into continuous activity, which made side-to-side alternation impossible to measure. Based on a concentration response experiment, we found that reducing the concentration of NMDA to 50 µM produced fictive locomotion where coordination could be assessed effectively, and therefore we used this lower NMDA concentration for all strychnine experiments. Strychnine (1 µM) significantly changed the properties of 50 µM NMDA-evoked fictive swimming in spinalized larvae without disrupting the episodic nature of the motor output (*n* = 6; Fig. 6). Episode frequency was significantly increased by strychnine while episode duration was significantly reduced (One-way repeated measures ANOVA; all *F*_(2,10)_>8.7; all *p*<0.006; Fig. 6C,D). Changes in episode frequency and duration were not reversed following washout (>30 minutes). Burst frequency and burst duration were reversibly increased by strychnine (One-way repeated measures ANOVAs; all *F*_(2,10)_>6; all *p*<0.02; Fig. 6E,F).

**Figure 7:**
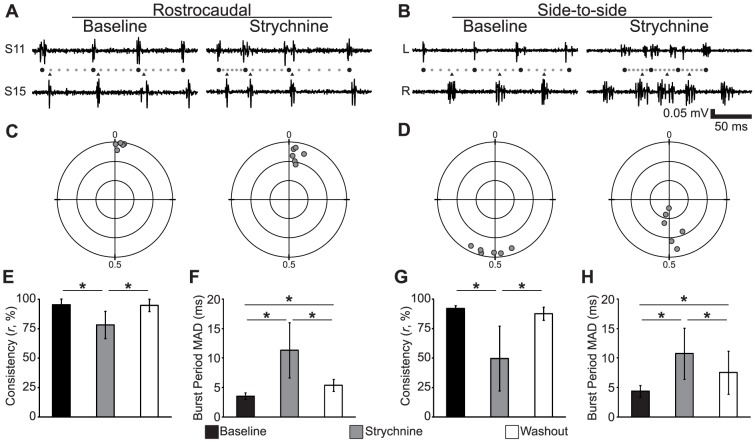
Reduced Synaptic Inhibition Reversibly Reduces Both Rostrocaudal and Side-to-Side Phase Consistency. (A–B) Representative traces of fictive motor activity recorded on the same side of the spinalized larvae in different segments (A) or on opposite sides of the larva in the same segment (B). Within each panel, left traces are activity at baseline (50 µM NMDA) and right traces are the activity in the same larva following addition of 1 µM strychnine. (A) The relative phases of the bursts in the paired recordings are illustrated using circles (phase markers) and triangles (follower burst times). Black circles indicate burst times, smaller gray circles divide each burst period into 6 equally long intervals. (C–D) Polar plots of the normalized phase vector sum (gray circles) of each preparation in the experimental group indicated above the plot. Concentric circles are plotted at distances of 0.33, 0.66 and 1.0 from the fixed point; cross-hairs separate the quadrants. (E–F) Plots of mean phase consistency (*r*) and mean burst period MAD against condition for ipsilateral recordings. (G–H) Plots of mean phase consistency (*r*) and mean burst period MAD against condition for contralateral recordings. * Statistically significant difference.

We also found that strychnine had significant effects on both phase consistency and motor rhythm stability (Fig. 7). Examining the traces of rostrocaudal delay in these spinalized preparations before and after application of strychnine did not reveal an obvious coordination defect (*n* = 6; Fig. 7A), however the vector sum analysis revealed a significant decrease in rostrocaudal phase consistency (One-way repeated measures ANOVA; *F*_(2,10)_ = 16.7; *p*<0.001; Fig 7C,E). There were more obvious changes in the burst structure of side-to-side alternation in strychnine, such as the overlap of bursts on contralateral sides of the body (*n* = 6; Fig. 7B). Strychnine caused a significant decrease in side-to-side phase consistency (One-way repeated measures ANOVA; *F*_(2,10)_ = 15.0; *p*<0.001; Fig. 6D,G). There were no significant differences in phase consistency between the baseline and washout (Corrected *t*-tests; all *t*<0.52; all *p*>0.62). The significant decreases in phase consistency of rostrocaudal delay and side-to-side alternation were accompanied by significant increases in burst period MAD (One-way repeated measures ANOVAs; all *F*_(2,10)_>10.9; all *p*<0.003; Fig. 7F,H). Strychnine had no significant effect on the mean rostrocaudal phase delay per segment or the mean phase of side-to-side alternation (One-way repeated measures ANOVAs; all *F*_(2,10)_<2.7; all *p*>0.11). Reducing inhibitory neurotransmission in spinalized larval zebrafish decreased both the phase consistency and stability of the motor rhythm, an effect like that spinal transections, but without disrupting episodic organization.

### Rostrocaudal Coordination is Impaired by Reduced Inhibitory Neurotransmission Independent of Motor Rhythm Stability

**Figure 8:**
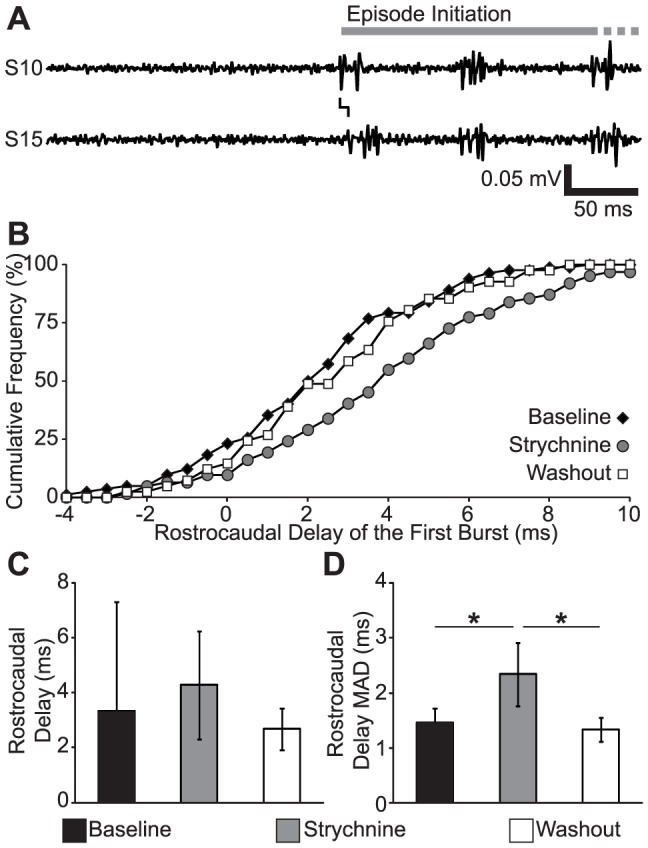
Reduced Synaptic Inhibition Significantly Reduces the Consistency of the Rostrocaudal Delay of Episode Initiation. (A) Representative traces showing the initiation of an episode of fictive swimming in Baseline conditions (spinalized, 50 µM NMDA). The gray bar shows the time in the trace occupied by the episode, the line between the traces shows the rostrocaudal delay between the first burst in the PN recordings. This episode continued past the time window shown in the trace, reflected by gray dots. (B) Cumulative histogram of rostrocaudal delay for a representative preparation in Baseline (black diamonds), Strychnine (gray circles; 50 µM NMDA, 1 µM strychnine) and Washout (white squares; 50 µM NMDA) conditions. (C–D) Plots of the mean rostrocaudal delay (C) and the MAD of rostrocaudal delay (D) against condition. * Statistically significant difference.

Reduced inhibitory neurotransmission resulted in the concurrent disruption of coordination and the stability of the fictive motor rhythm. In order to exclude the effect of an unstable motor rhythm as the cause of the decreased coordination, we considered a situation where locomotor bursts are produced in the absence of an ongoing locomotor rhythm: the first burst of each episode of fictive swimming (Fig. 8A). The distribution of first burst rostrocaudal delays appeared to be broader in strychnine than at baseline or washout (Fig. 8B). We quantified variability of the first burst delay in the time domain by measuring the mean rostrocaudal delay and the MAD of the delay (Fig. 8C,D). There was no significant effect of strychnine on the mean rostrocaudal delay of the first burst of each episode (One-way repeated measures ANOVA; *F*_(2,10)_ = 0.94; *p* = 0.42; Fig. 8C). There was a significant effect of strychnine on the MAD of the delay of the first burst of each episode (One-way repeated measures ANOVA; *F*_(2,10)_ = 17.9; *p*<0.001; Fig. 8D), due to increased variability in the strychnine condition (Corrected *t*-tests; all *t*>4.8; all *p*<0.001). There was no significant difference in rostrocaudal delay MAD between the baseline and washout (Corrected *t*-test; *t* = 0.68; all *p* = 0.51). Both the first-bust analysis and the phase vector sum of all bursts show the same effect of strychnine: decreasing the consistency of locomotor coordination. Because the disruption of rostrocaudal delay by strychnine is found in the absence of an on-going locomotor rhythm we conclude that the perturbation is not caused by an unstable locomotor rhythm, and therefore that strychnine disrupts coordination independent of disrupting episodic organization.

## Discussion

In this study we characterized the coordination of fictive motor activity produced by intact and perturbed larval zebrafish spinal cords. In spinalized preparations, this fictive motor output could be accurately described as “fictive swimming” because it retained the characteristics of larval zebrafish locomotion: episodic organization and coordination [39], [40](Figs. 1–4). Fictive motor output in reduced spinal cord preparations lacked episodic organization, and both the stability of the motor rhythm and the consistency of rostrocaudal and side-to-side phase relationships were reduced (Figs. 1–4). Reduced phase consistency would make muscle forces add less efficiently on a cycle-by-cycle basis, weakening each tail stroke and likely impeding important survival behaviors. Therefore, we argue that phase consistency captures a behaviorally relevant aspect of swimming that is impacted by our experimental manipulations. Neither the lack of episodes nor the disruption of the motor rhythm was responsible for the decreased phase consistency (Figs. 7,8). This result is also not solely a function of the level of overall excitation, since 100 µM NMDA drove bursting at equal frequencies in all preparations (Fig. 2); a good proxy measurement for global excitation [29]. Instead, the transections appear to have directly disrupted the circuits controlling rostrocaudal delay and side-to-side alternation.

### Coordination Relies on a Non-Segmental Circuit

The primary goal of these experiments was to determine which of two competing models (segmental CPGs or continuous gradient) of the spinal locomotor circuit better describe the larval zebrafish spinal cord. In previous work, we demonstrated that rostrocaudal and side-to-side coordination were not impaired in reduced preparations of 12 or more spinal segments [29]. Therefore we have not repeated these experiments, and in this report we have focused on transections that spare fewer (10 or 5) spinal segments in order to determine their effect on coordination. These transection experiments demonstrated that fictive motor coordination diminishes as the number of intact spinal segments decreases (Figs. 3,4).

Rostrocaudal coordination between two adjacent segments does not depend solely on direct connections between the segments, and side-to-side coordination does not solely depend upon commissural connections between opposing hemi-segments. Instead, both rostrocaudal and side-to-side coordination depend on how much of the surrounding spinal cord is intact. This finding is especially surprising for side-to-side alternation, a process that could plausibly take place completely within a segment. The segmental CPG model could explain this data if each segmental CPG were perturbed so that they produced an unstable motor rhythm and intra-segmental coupling were too weak to entrain the outputs. Exploring this alternative hypothesis, we found that spinal transection compromised the stability of the motor rhythm (Figs. 3,4). However, we also found that spinal transections reduce phase consistency within hemi-segments (Fig. 5), which would not be predicted by a segmental model even when rhythm stability is compromised.

Based on these results, we conclude that the spinal locomotor network of the larval zebrafish is unlikely to consist of segmental CPGs. The reduction of coordination we observed is better explained by an unsegmented locomotor system, similar to models of the tadpole locomotor CPG [20]. In the computational model of the tadpole locomotor system, rostrocaudal delay of motor neuron firing results from a non-segmental gradient of descending excitatory neurons and their synapses [20]. Removing the excitatory gradient and making the neuronal/synaptic density equal along the spinal cord results in a loss of rostrocaudal delay [20]. Using this paradigm, one would predict that a transection that removed many of these projection neurons from the circuit would reduce the strength of the synaptic gradient necessary to maintain a rostrocaudal delay.

The loss of side-to-side coordination that we observed following spinal transections (Fig. 4) was not predicted by either the segmental or a gradient-driven model of the locomotor system [7], [20]. We hypothesize that the loss of side-to-side alternation following transection is due to the loss of commissural inhibition from neurons distributed throughout the spinal cord [41], [42]. There are four identified classes of commissural inhibitory interneurons in the larval zebrafish, of which only the CoSA and CoBL neurons are active during swimming [43]. Both of these neuronal classes have long-range projections (∼10 body segments) that would be disrupted by spinal transections. We hypothesize that even through CoBL neurons are relatively abundant, the overlapping projection fields of CoSAs and CoBLs distributed throughout the spinal cord are necessary to produce consistent side-to-side alternation.

### Episode Generation and Coordination are Independent

One limitation of spinal transection experiments is that the degree of transection necessary to disrupt coordination also disrupts episode generation (Fig. 2; see also [29]). Consistent with previous findings [24], [44], disrupting glycinergic neurotransmission does not disrupt episodic organization (Fig. 6). We found that strychnine has the effect of reducing the stability of the locomotor rhythm and reducing phase consistency of spinalized fictive motor output (Fig. 7). The coordination deficit revealed by phase vector addition of all bursts was recapitulated in an analysis of only the first burst of each episode, which eliminates the disruption of the locomotor rhythm as the cause of reduced coordination (Fig. 8). These results confirm and extend the results of our previous report [29], demonstrating that there is a dissociation between episodic organization and coordination. One caveat to these findings is the possibility that homeostatic processes following the elimination of inhibition may unmask mechanisms different from those that ordinarily drive locomotion [45], but we have no direct evidence that this is the case. A possible future direction of this research would be to use optogenetic tools (eg, ArchT [46]) to synaptically isolate regions of the spinal cord reversibly and with greater spatial resolution. An optogenetic approach, in addition to greater precision, would allow cell type selectivity based on neurotransmitter profile or projection pattern [44], [47], and may reveal the topography of functional networks.

### Conclusions

Our findings are inconsistent with larval zebrafish having a segmented locomotor CPG. These findings make it difficult to sustain the body segment as an important feature of the larval zebrafish locomotor system in the organization of the pre-motor network. Instead, both the present study and recent work on larval zebrafish pre-motor interneurons [36], [44], [48] suggest that the larval zebrafish CPG is an unsegmented network of microcircuits. Short-range connections between interneurons and from interneurons to motor neurons are almost certainly important features of this network, but the utility of describing these connections as “intra-segmental” is unclear.

We hypothesize that the larval zebrafish locomotor CPG is functionally segregated as follows: Episodes of locomotion are initiated and maintained by synaptic drive originating in the V2a neurons of the hindbrain [49], [50]. The spinal cord, despite its inability to initiate or maintain a locomotor episode, has an episode termination mechanism, demonstrated by the termination of episodes in spinalized larvae [28] and the modulation of episode duration at the spinal level [35]. The episode termination mechanism: 1) requires>12 intact segments of the spinal cord to effectively suppress motor output [29], 2) has better performance when the circuit is strongly excited [29], and 3) does not require glycinergic neurotransmission (Fig. 6). Independent of episode generation, there are a collection of microcircuits that drive rhythmic motor output with appropriate phase relationships along the larvae. The coordination circuit is distributed throughout the spinal cord and requires ∼10 intact segments to perform well (Fig. 3,4). The coordination circuit requires excitatory and inhibitory neurotransmission (Fig. 7). Spinal V2a neurons are likely one source of excitation in the coordination circuit [36], [44], and inhibition is likely supplied by both ipsilateral and contralateral projection neurons [38], [43].

The functional dissociation we find between burst period variability and coordination mirror functional dissociations of these properties between genetically defined ventral excitatory interneurons in the mouse spinal CPG [51]. It is unclear to what degree the larval zebrafish and mouse CPGs share spatial organization. In the hindlimb region of the spinal cord, the properties of locomotor deletions are well explained by ipsilateral pattern-forming networks that coordinate the activity of flexor and extensor motor pools [22], [52]. In contrast to an interdependent flexor-extensor network, some recent data supports the existence of independent unit burst generators (UBGs) located adjacent to their motor neuron outputs [53]. Even if the UBG hypothesis is correct in the lumbar region, concieving of the mamalian spinal cord as a collection of equipotent UBGs distributed along the spinal cord would be an oversimplification [54]. In contrast to a UBG architecture, we did not find evidence for coordination circuits adjacent to their axial motor output in the larval zebrafish. However, the finding that motor neuron activity is produced in all of our reduced preparations (Fig. 2) suggests that it is possible that localized motor circuits exist for functions other than episode generation and coordination. Regardless of the degree of conservation of specific neuronal structures between zebrafish and other animals, cross species comparisons are often instrumental in developing deeper understanding of each system [55].
